# On the Use of Metal Sinter Powder in Laser Powder Bed Fusion Processing (PBF-LB/M)

**DOI:** 10.3390/ma16165697

**Published:** 2023-08-19

**Authors:** Jan-Simeon Ludger Bernsmann, Simon Hillebrandt, Max Rommerskirchen, Sebastian Bold, Johannes Henrich Schleifenbaum

**Affiliations:** Digital Additive Production DAP, RWTH Aachen University, 52074 Aachen, Germany; simon.hillebrandt@rwth-aachen.de (S.H.); sebastian.bold@dap.rwth-aachen.de (S.B.); johannes.henrich.schleifenbaum@dap.rwth-aachen.de (J.H.S.)

**Keywords:** PBF-LB/M, best cost, low cost, laser beam source, motion system, sinter powder

## Abstract

Metal Laser Powder Bed Fusion (PBF-LB/M Powder Bed Fusion, Laser-Based/Metal) offers decisive advantages over conventional manufacturing processes. Complex geometries can be produced that cannot, or only to a limited extent, be manufactured with conventional manufacturing processes. One of the main disadvantages of the process are high investment and operating costs. In order to make the PBF-LB/M process accessible to new research areas, the costs need to be reduced. Therefore, this work investigates whether laser beam sources and motion systems in currently established PBF-LB/M systems can be replaced by more cost-effective components. To reduce the operating costs for PBF-LB/M, the studies are carried out based on previous work with water-atomized, process-foreign sinter powder instead of gas-atomized, spherical PBF-LB/M powders. A cost-efficient, low-alloyed powder is selected (Höganäs HP1) and processed on two different PBF-LB/M machines with a restricted process window using process parameter values that current low-cost machines can achieve. The results show that a multimode fiber laser leads to a more stable process and wider melt pools compared to a single mode fiber laser. In addition, a lower sensitivity of the process with respect to modified process parameters is observed for the multimode laser, resulting in a wider range of stable process windows. A Cartesian motion system (gantry) is suitable for use in PBF-LB/M despite lower scan speeds compared to galvanometer scanners. Beam guidance in the XY-plane offers new possibilities for machine and process design that are not possible with usual scanner systems.

## 1. Introduction

In order to make the PBF-LB/M process economically viable even for occasional applications in small and medium-sized enterprises (SMEs), whose primary field of work is not additive manufacturing, investment and operating costs need to be reduced. The PBF-LB/M process is expensive for SMEs due to the acquisition costs of the necessary system technology, which can quickly exceed EUR 500,000, running costs to operate the equipment as well as a lack of know-how to use the technology [1]. There are two main cost drivers within the PBF-LB/M processes: the machine cost and the material cost. An investigation of the main cost drivers in PBF-LB/M concludes that 73% of the manufacturing costs of a component are caused by the investment costs in the system technology, while the material costs make up a non-negligible 11% [2]. The machines themselves are typically equipped with expensive high-quality lasers as a radiation source, because low-beam-quality lasers lead to challenges in focusing and forming stable melt pool geometries. A further possible component for cost reduction within the machines are the motion and shaping systems that focus the laser beam and guide it on the print bed for hatch and contour generation [3]. An alternative low-cost solution are gantry systems; however, these lead to particular limits on the speed at which the laser beam can be guided. Due to its high costs, PBF-LB/M technology is only economical for industrial serial production if the systems are used under high machine capacity utilization. Concerning the material cost, there exists a large body of research work on different methods of powder atomization to reduce powder cost. However, this work is often only targeted at high-alloy materials as machine costs make low-alloy materials uneconomical. However, steadily falling machine costs are making low-alloy materials in these processes increasingly attractive, especially to address new sectors and fields of application such as the craft or the construction industries. This work therefore investigates the transferability of two different PBF-LB/M machines using a comparably inexpensive powder [4]. A novel approach is to replace powders specialized for PBF-LB/M processes by widespread process-foreign powders, e.g., from sintering.

This work addresses the material cost factor side. In general, PBF-LB/M powders are tailored to specific application areas in which the process is already established. The requirements of the material are very high, especially in the aerospace industry and medical technology, and can often only be met by advanced materials. The acquisition costs of PBF-LB/M powder can therefore often be 10 to 20 times higher than those of metal powders used in other common processes such as powder coating or sintering. Moreover, in sophisticated industrial plants, it is not profitable to operate with powders whose properties limit the potential performance of the plant.

A preliminary market analysis carried out shows that PBF-LB/M machines are predominantly equipped with single-mode fiber lasers as a beam source and galvanometer scanners for beam guidance. To reduce system costs, the first low-cost PBF-LB/M concepts used a cheaper diode laser instead of a multimode fiber laser as well as a cartesian motion system (gantry), which eliminates the need for expensive optics. For this purpose, multiple builds were processed both on a usual PBF-LB/M system and on a low-cost machine, based on the requirements and possibilities of SMEs and craft businesses [5]. The properties of the samples produced were compared using various metallographic analysis methods.

## 2. Materials and Methods

### 2.1. PBF-LB/M Machines

Cubed samples with a surface of 5 × 5 mm^2^ and a height of 12 mm were manufactured using the Aconity 3D MINI PBF-LB/M machine (from the Aconity3D GmbH, Aachen, Germany [6]), which is equipped with a single mode fiber laser with a maximum laser power (P_L_) of 400 W from IPG Photonics, a scanner system with an F-Theta lens (f = 420 mm, λ = 1030–1080 nm) resulting in a laser spot diameter of 140 μm (defocused). These specimens were also manufactured using an ALPHA 140 (Laser Melting Innovations GmbH, Aachen, Germany [5]). The system is equipped with a 200 W multimode fiber laser and a gantry consisting of linear guides, ball screws and servo motors. This enables scanning speeds (v_s_) of up to 150 mm/s. The laser emits radiation with a wavelength of 1070 nm and the focus diameter is 140 µm. For both studies, a build platform with a diameter of 140 mm is used. Regarding the scanning strategy, a bidirectional *XY*, meandering, scanning path with 90° rotation on each layer was used during the builds (individual AM-builds).

### 2.2. Materials

#### 2.2.1. Costs

Expensive alloying elements such as molybdenum, vanadium, nickel or beryllium as well as cost-intensive manufacturing processes increase the purchase price of a metal powder. In order to keep the purchase cost as low as possible, steels that contain no or the lowest possible content of expensive alloying elements and are produced by inexpensive manufacturing processes such as water atomization are used [7,8].

#### 2.2.2. Powder Handling/Occupational Safety

Particle-size distributions (PSDs) of metal powders designed for PBF-LB/M are wide in intervals from 15 to 45 µm. Handling of powders with this PSD may be hazardous and is usually carried out within the process periphery such as closed gloveboxes, handling stations and sieve-stations under extracted air conditions to ensure safety for the operator. These devices are not generally available to SMEs, especially for those who want to establish AM processes in their business. To facilitate handling of the powder, care is taken to ensure that the smallest particle size is above the alveolar range. According to EN 481, particles with an aerodynamic particle diameter *D*_AE_ ≥ 16 μm are no longer alveolar. The alveolar fraction are particles small enough to penetrate the alveoli. To ensure that there were no alveolar undersize particles in the powder, the powder batches were sieved with a higher particle size of at least 45 µm up to 90 µm. In order to compare the effects of the PSD on the relative density, a second powder batch was created in which grains smaller than 90 µm were sieved [9,10].

#### 2.2.3. Powder Selection

All specimens were manufactured with the metal powder DISTALOY HP1 from the manufacturer HÖGANÄS located in Höganäs, Sweden [11]. The material used represents a low-alloy steel that would be typically used in SME environments for inexpensive parts, which are welded together in a conventional manner out of inexpensive semi-finished products such as sheet metal and pipes. The sinter powder used in this work is 95% cheaper than a 1.4404 PBF-LB/M-powder [12]. The powder is a low-alloy iron-based powder specially developed for the sintering process. The composition of this HP1 powder is shown in Table 1.

The cost of the powder used was about EUR 3/kg (2022). At a yield of 30% after sieving of the powder, the cost corresponds to about 1/3 of the cost of a high-alloy PBF-LB/M metal powder of 1.4404. The powder selected for the investigations is a sintered powder. As an iron-based powder, it largely consists of favorable alloying elements. Due to the widespread use of the sintering process, it is ensured that the powder is quickly available.

**Figure 1:**
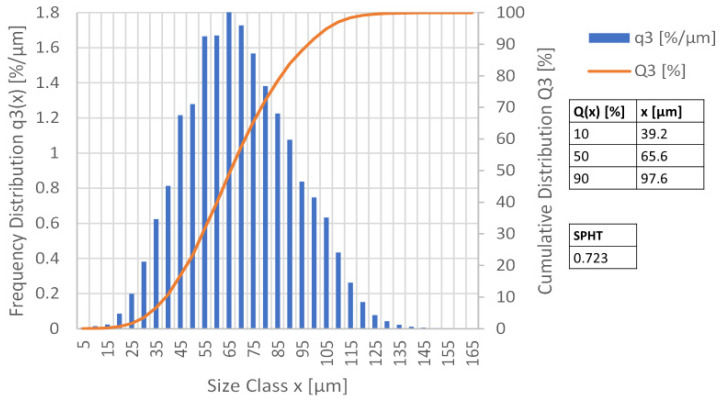
Example PSD Powder analysis of the 0–90 µm HP1 Batch used in Build 5–6 with frequency and cumulative distribution. The volume-related sphericity (SPHT) was also determined. This describes how uniform the surface texture and shape of a particle is. Perfect spheres have a sphericity of 1. Achievable relative densities and mechanical properties of the components increase with the sphericity of the powder used The mean value of the sphericity of the powder used was measured at SPHT = 0.723.

The powder selected for the studies was divided into batches. For this purpose, the limits of the grain sizes were conservatively selected based on the specifications from EN 481 at a grain size above 45 µm. The criteria defined for the selection of a suitable powder are all fulfilled by the selected HP1 sintering powder. In Figure 1 the PSD powder analysis of one powder batch is shown. The frequency distribution q(x) of the particle sizes is shown as a bar chart. From it, the proportion of particles with a fixed particle size can be taken. In addition, the cumulative distribution Q(x) is shown. The cumulative distribution indicates the proportion of all particles with a particle size less than or equal to a fixed value and is derived from the first derivative of the frequency distribution. Consequently, it can be taken from the PSD that half of all powder particles are smaller than 65.6 µm. 90% of all particles are smaller than 97.6 µm.

**Figure 2:**
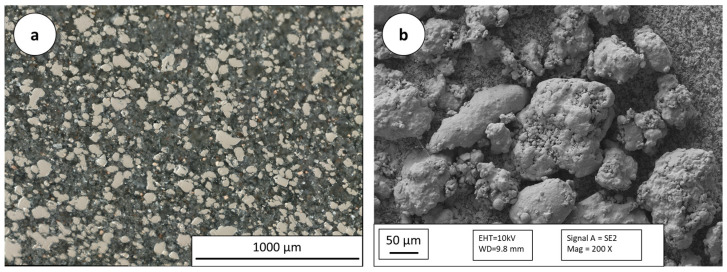
OM image of the embedded powder (**a**), SEM image of the powder (**b**).

Specimens were built up with the batch of grain sizes of 45–90 µm. The powder is water atomized, so that the powder has a non-spherical morphology (see Figure 2).

The grain sizes are clearly above the thoracic fraction. In addition, no highly reactive substances, which would complicate powder handling, are contained in the selected powder. In the comparative test with a batch with particle sizes < 45 µm, no increase in the mechanical properties could be achieved. The target aspect of work safety and powder handling is fulfilled.

#### 2.2.4. Process Parameters and Hatch Strategy

All samples were manufactured on the PBF-LB/M systems “Aconity 3D MINI” (MINI) and “ALPHA 140” (ALPHA). The inert gas used in the MINI was argon whereas nitrogen was used in ALPHA 140. Although the MINI system is equipped with a fiber laser with a scanner system, the process parameters were set to the volume energy and buildup rate corresponding to the values achievable with low performance diode laser and gantry equipped systems.

A small process window presented in Table 2 was pre-defined. These limits result from the characteristic values of low-cost diode lasers, beam guidance and collimation as well as the characteristic limits on motion of low cost gantry systems for which the parameters are designed [6]. The positioning accuracy of the used gantry especially causes a high hatch distance when compared to the MINI.

A bi-directional exposure for all specimens was used with 90° rotation and all specimens were rotated by 45° against the shielding gas flow. Build 1 Substrate plate with specimens (a), aborted exposure of specimens in red (b)). The coating and exposure procedure was only doubled in build 4. The powder batches used for the individual builds (# represents number) are shown in (ref. Table 3).

When varying the scanning speed in these, when compared to literature values, low intervals, Simchi and Rombouts observed a tendency towards higher relative density at a lower scanning speed. Therefore, a lower scan speed of 50 mm/s was chosen and increased in steps of 50 mm/s to a maximum of 150 mm/s [13,14].

The layer thickness was set to 100 µm. In the above-mentioned studies, layer thicknesses between 70 µm and 200 µm were investigated. The hatch distance (y_s_) was varied in steps of 50 µm between 100 µm and 300 µm. A process parameter validation was executed on the MINI and then adapted for the ALPHA.

#### 2.2.5. Density Analysis

**Figure 3:**
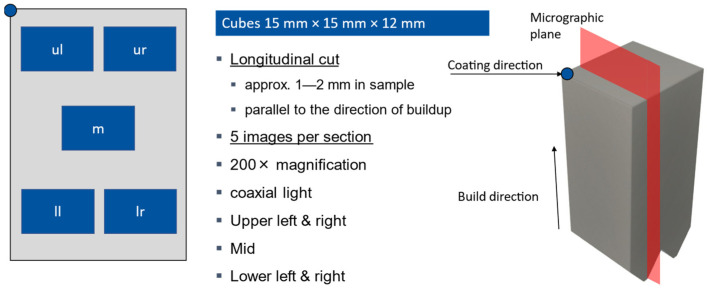
Schematic of determining the rel. density according to VDI 3405 [15].

The relative density was determined according to VDI 3405 [15] with image porosity analysis software using light microscope images of specimen cross-sections (see Figure 3). For this purpose, five close-up images were taken, distributed over the total area of a cross-section. The average relative density was then calculated for each specimen [15,16].

With this method, it is possible to determine the defect types in particular and to obtain an initial overview of the relative density without having to use CT scans.

All subsequent values of the relative density refer explicitly to this representative level.

#### 2.2.6. Particle Size Distribution

To determine the particle size distribution (PSD) after sieving the fine and coarse grains from the original powder batch, powder samples were measured with a Camsizer X2 (Microtrac Retsch GmbH, Haan, Germany). The device determines the particle size distribution by taking pictures of a measuring field on which the particles of the sample have previously been dispersed.

## 3. Results

The variations of powder batches in their PSDs, process parameters, hatch strategy, exposure and coating (Ex&Co) as well as the equipment used are summarized in Table 1.

The results of the individual builds are shown in the following sections:

### 3.1. Build 1–3, ACONITY 3D MINI

**Figure 4:**
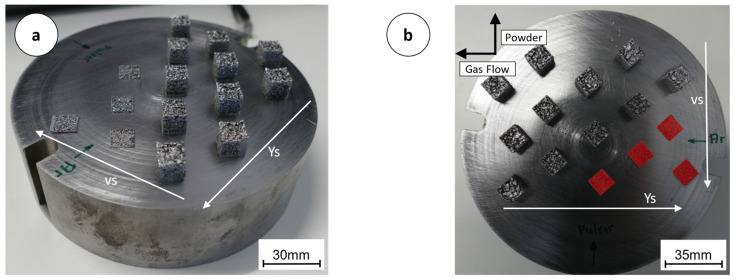
Build 1 substrate plate with specimens (**a**), aborted exposure of specimens in red (**b**).

The surface of the cubes of the first build was porous and the upper surface of the cubes were caved in. While welding, the air-filled spaces in the powder-bed are filled by the melt. The height of the solidified layer decreases significantly compared to the height of the gas-atomized powders manufactured for PBF-LB/M. After removing loose powder particles from the finished cubes, a very open-pored structure was visible over the entire cross-section of all specimens, and light passed through the cavities within the components. In Figure 4, the substrate plate with the built-up samples is shown. Particularly for the samples which were produced with a low volumetric energy density, it is noticeable that hardly any powder was melted and built a fusion–metallurgical bond. Shape retention and filling of the specimens decreases with increasing scanning speed and hatch distance. Within the first build, 11 out of 15 specimens were successfully built, albeit with the described porosity. To avoid damage to the coater by protruding tips and edges of the affected cubes, the exposure of four specimens was aborted. This is true for those specimens with the highest hatch distance and highest scanning speed within build 1, Figure 4. In Figure 4, the corresponding cubes are marked in red. In addition, the directions for powder application (v_s_) and the exposure sequence (y_s_) are shown. The build direction (BD) was orthogonal to the substrate plate. The shielding gas flow is also indicated in Figure 4.

The metallurgical bond between the specimens and substrate plate was comparably low due to their high porosity, allowing for an easy removal from the substrate plate with forceps. Since it was already visually obvious that the specimens were highly porous, no quantitative examination was performed. During the process, individual solidified spatters lying on the powder surface were clearly visible. When the lid of the system was opened after the process finished, dark powder areas were visible to the side of the supply cylinder. This could be observed in scuff marks and spatters, which were deposited on the powder surface and led also to insufficient recoating during the process. Especially in the backwards movement, the coater drags these particles and thus draws them towards the supply cylinder.

**Figure 5:**
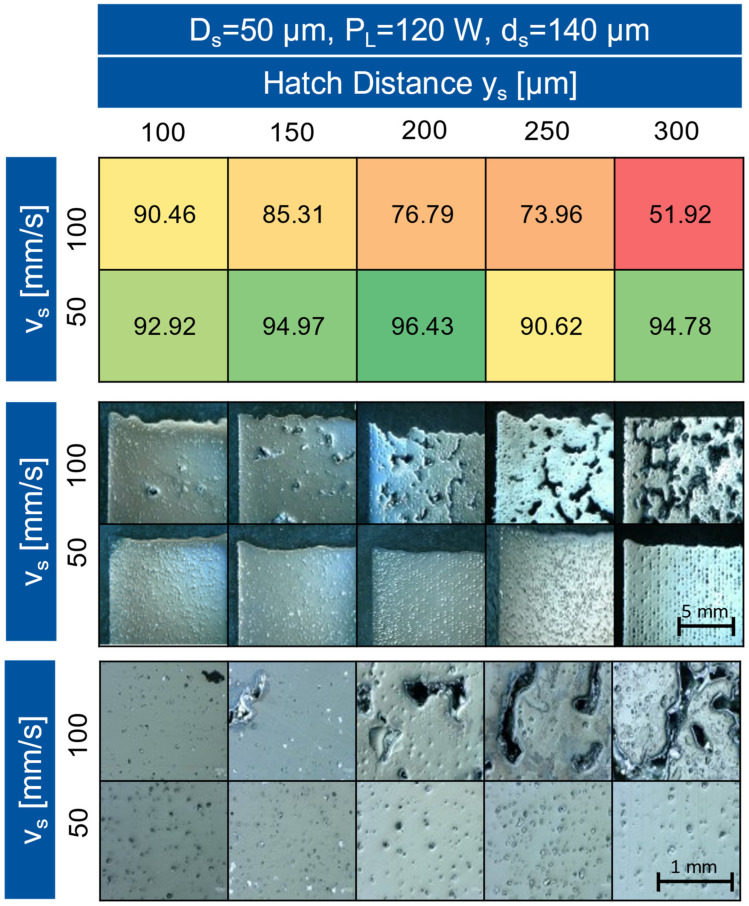
Microscope images with variant mag. of the build 2 for two different scanning speeds.

For the following builds, the supply factor was increased from 1.5 to 2. The layer thickness D_s_ was lowered to 50 µm and the laser spot diameter was increased to 140 µm by defocusing. The aim was to achieve a state of equilibrium in which the thickness of the solidified layer corresponds to D_s_, the distance by which the substrate plate descends in each cycle run, defined in the process parameters. The excess powder in the coatings counteracts the collapse of the cubes, so that all cubes could be built without complications. In particular, the cubes built at a high scanning speed have a foam structure and contain large pores with diameters up to 300 μm. It is noticeable that slower scanning speeds produced more uniform and dense surfaces. With increasing hatch distance, the surface of the cubes became more uniform, and the dimensional stability of the cubes increased. The specimen contours and the detailed cross-sections of the cubes are shown in the microscope images in Figure 5.

In direct comparison, it is noticeable that samples with a slower scanning speed contained less but significantly larger inclusions than specimens that were built up at a high scanning speed. The areas that appear dark in the images are cavities in the specimen filled with embedding material. During the grinding of the specimens for the microscope images, powder particles that were not melted in the process were released from the cubes and made the preparation challenging (Figure 5). Due to the size and the rough contours, which are untypical for pores, the cavities in the cubes that appear black in the images are spaces that were previously filled with powder particles that were not melted during the manufacturing process. The occurrence of large inclusions with angular contours is typical for powders of low sphericity due to the strong tendency to form agglomerates. With increasing volumetric energy density, the effect occurs to a lesser extent as shown in Figure 5 [17].

The shape retention of the sample, which was built up with a hatch distance of 200 μm and a scanning speed of 50 mm/s, was the highest in comparison to the remaining samples of the build. At both higher and lower hatch distances, the cubes collapsed more, and the surface became less uniform. In the micrographs, these effects of collapsed cubes are visible as small shiny dots with angular outlines. The powder inclusions form the largest part of the defects occurring in build 2. While the defects were randomly distributed in the sample at low hatch distances, the defects formed a uniform linear structure at high hatch distances. Due to the large hatch distance and the resulting smaller overlap of the tracks, areas are created in which the temperature is lower due to the lower energy density. In these areas, the powder cannot be completely melted [14,18].

Based on the trends from builds 1–2, scanning speeds between 50 mm/s and 100 mm/s were investigated in small steps in the following builds. In order to completely melt the powder spill, a higher laser power of 150 W was included in the parameter space as an additional parameter. Inclusions with pointed contours can cause a drop in mechanical strength. When force is applied, stress peaks form at the edges, which can cause cracks to form. Therefore, the parameters were primarily varied with the intention of completely melting all powder particles.

In the microscope images of the samples from build 3, which were built up with 150 W laser power, fewer powder inclusions are visible compared to the samples built up with a laser power of 120 W. The higher laser power ensures more complete melting of the powder particles. However, there were still inclusions of non-melted particles in all samples. The size of the powder inclusions increases with increasing laser power. At a high volume energy density, the structure of the material in the micrographs is more homogeneous between the large inclusions. With a higher energy input, greater melt pool spreading is expected. To prevent overheating of the melt due to too high overlap of the tracks, the hatch distance was increased. The build-up of samples with a low scan speed and small hatch distance takes a long time compared to conventional PBF-LB/M parameters. If the number of tracks per layer becomes smaller, the build-up time also decreases, making the process more economical. The supply factor was changed to 3 in each cycle to further counteract cube collapse.

**Figure 6:**
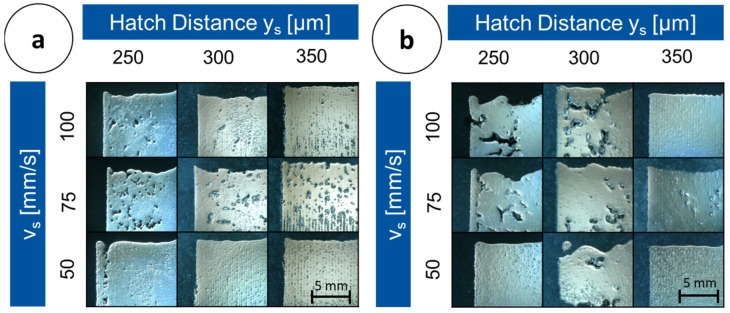
Microscope images of the sample contour build 3 ((**a**) P_L_ = 120 W, (**b**) P_L_ = 150 W).

Figure 6 shows the contour of the specimens built up with 120 W and 150 W laser power in comparison. In the images of the specimens built up with a high laser power, clearer differences between the different micrographs can be seen. For the samples built with y_s_ = 250 mm/s and Pl = 120 W, the number of inclusions and defects decreases slightly with decreasing scan speed and the inclusions become smaller. The sample built with Pl = 150 W, y_s_ = 250 µm and y_s_ = 150 mm/s contains numerous large inclusions, whereas the sample built with Pl = 150 W, y_s_ = 250 μm and v_s_ = 50 mm/s contains significantly fewer visible defects. Especially the samples which were built up with the parameters Pl = 150 W, v_s_ = 75–100 mm/s, y_s_ = 250–300 μm contained large inclusions and showed a non-uniform wavy surface. The non-uniform structure of the samples with a high scanning speed occurs due to the high viscosity of the melt at too low energy input. The surface tension is so high that the melt contracts into individual spherical points, as this is the most energetically favorable state.

This effect becomes visible in the form of the wavy surface structure and large inclusions in the samples. The measured density increases again with increasing lane spacing. At the same time, hardly any large inclusions occur. An increased occurrence of defects at too low a hatch distance also occurs in investigations by Meiners [19]. The effect occurs when the overlap of the laser beam with the already solidified tracks is so large that the mainly solidified material is partially remelted. The melt of the newly molten powder is deposited on the existing track. Due to the surface tension of the melt, the melt pool contracts. This results in a wavy upper surface of the cubes and voids between the welded tracks. When the subsequent layer of powder is applied, particles slide down and fill the void. If agglomerates form in the process, or if a large particle slips into a cavity, it cannot be completely melted and remains as an inclusion. If the hatch distance increases, a uniform structure of vertically superimposed defects is visible in the position of the defects. This effect is due to insufficient track overlap. Since a higher laser power leads to a larger melt pool spread, the effect occurs more often with low laser power samples than with high laser power samples. If the scanning speed is reduced, the effect also occurs to a lesser extent.

The relative density is within the range achieved by comparable studies (see 2.2.5). It lies between 81.66% and 95.71%. In a direct comparison of the parameter combination, an increase in laser power leads to a higher relative density. The highest density achieved in build 3 of 95.71% was reached with P_L_ = 150 W. The density readings correlate with the amount of large defects. Due to their size of up to 300 μm, they significantly determine the measured value. The amount of large defects correlates with the standard deviation of the density measurements. In order to be able to make a statement about the microstructure between the large defects, the density is weighted with the standard deviation of the density measurements. With a higher laser power, higher density samples can be built up more quickly. Overall, a trend towards lower scan speeds and higher hatch distance is visible. In the parameter field with a laser power of 150 W absolute, the highest relative densities are achieved in the parameter field of build 3, both with the parameter combination with the highest volume energy density and with the parameter combination with the lowest volume energy density.

### 3.2. Build 4, ACONITY MINI

**Figure 7:**
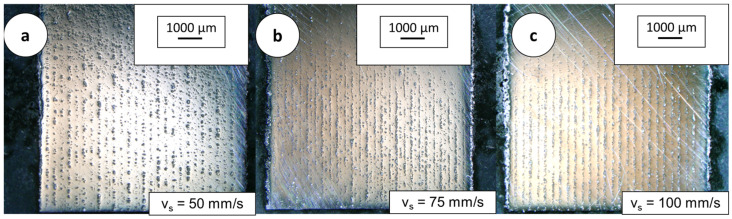
Linear defect arrangement (P_L_ = 120 W, y_s_ = 350 µm) observed in samples prepared with the double exposure/recoating strategy. (**a**) v_s_ = 50; (**b**) v_s_ = 75; (**c**) v_s_ = 100.

In this entire build, a process procedure was used in which every new powder layer was exposed, then recoated and exposed again without lowering the build platform. Since the laser power of the laser system of the low cost systems may be limited, it was not increased. Compared to the previous build, it is visible that the double exposure/recoating strategy can completely suppress the occurrence of large inclusions and defects. Pore formation was not visible. The only defects that did still occur were powder inclusions. The contour of most of the included particles was round, indicating that they could have been melted at least superficially or that spatters were deposited on the powder bed. The structure of the cubes of the different laser powers is fundamentally different. While at P_L_ = 120 W, the cubes that were processed with v_s_ = 50 mm/s had the most visible inclusions in the parameter field, at a higher laser power of P_L_ = 150 W the cubes that were built up with the highest scanning speeds showed the most inclusions. At a low laser power, a decrease in the amount and size of inclusions was visible with increasing scan speeds. The influence of the hatch distance on the amount and structure of the inclusions appeared to be small. For samples built at v_s_ = 75 mm/s, the arrangement of inclusions followed the hatch distance. At low scan speeds, there were so many inclusions that no structured arrangement was visible. At a high hatch distance, a non-uniform structure was formed over the height. Near the substrate plate, the cubes are crisscrossed by vertical lines of unfused powder residue. The melt contracts due to the high viscosity and associated high surface tension. Voids are formed between the traces of contracted melt, which are filled with powder during coating, shown in Figure 7. The powder enriched in the voids gradually detaches from the samples during sample preparation, so that the preparation of the samples and the parallel light orientating on the microscope is challenging (sample on the right, Figure 7).

**Figure 8:**
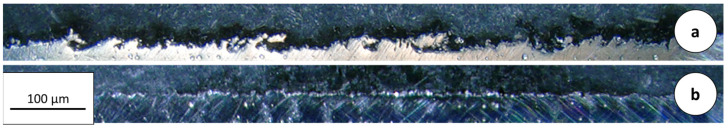
Inert gas flow averted (**a**) and facing (**b**) side of a transverse section.

At high laser powers, the amount and size of inclusions decreased with decreasing hatch distances. Especially at low scan speeds, the surface of the cubes was wavier than that of the cubes built with low laser power. The position of the defects followed a linear structure for all hatch distances. Over the height, the cubes showed a uniform structure of those defects. The contour of the cubes was significantly less collapsed than in the previous builds (1–3). It is noticeable that for the samples built with P_L_ = 120 W, y_s_ = 300 µm–250 µm, the side opposite the inert gas flow had a rougher surface than the side facing it. The effect is illustrated in Figure 8. The rough side surface is created when spatter from the shielding gas flow is carried to the side facing away from the flow, where it sinks and is deposited on the powder bed. The spatter particles are larger than the powder particles. They are not completely melted and a rough, unclean edge results [19,20].

**Figure 9:**
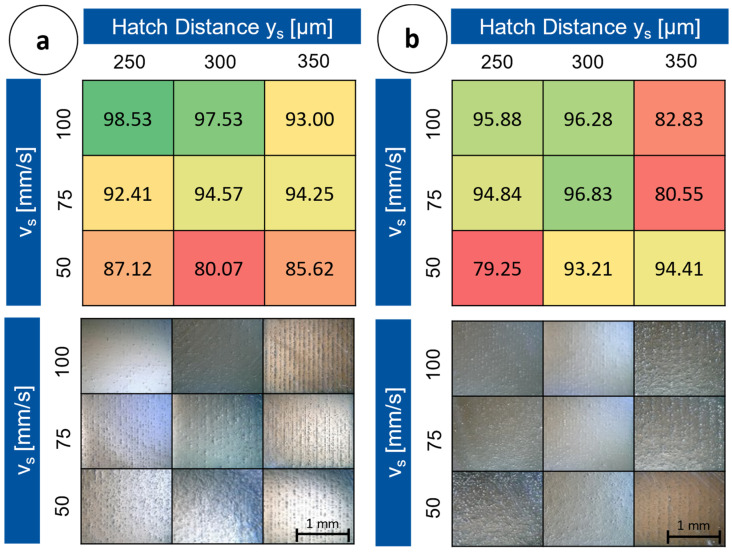
Micrographs and rel dens. from build 4, P_L_ = 120 W (**a**),and P_L_ = 150 W (**b**) double exposed.

The maximum relative density could be increased to 98.53%. To address the defects especially within the samples built with high scanning speeds, which contained a lot of inclusions compared to the samples with a lower scanning speed, the coating and exposure procedure was doubled per layer. In contrast to the single-exposed specimens (build 1–3), higher density values were achieved with double exposure at a lower laser power. Compared to the density values of the singly exposed samples, only a trend of increasing densities towards higher scan speeds and lower track gaps is visible. The density values correlated with the number of defects visible on the microscope images. In Figure 9a,b, the density values with micrographs of the corresponding specimens are shown.

The density readings of the samples built up with a laser power of P_L_ = 150 W decreased from the specimens built up with the parameters v_s_ = 75 mm/s and y_s_ = 300 µm. With increasing laser power, the relative density dropped in some cases.

### 3.3. Build 5, ACONITY MINI, and build 6, LMI ALPHA 140

In order to validate whether an improvement in the shape retention and density of the specimens can be achieved with a wider PSD, specimens were also built from a powder batch in the interval of 0–90 μm. Due to the lower porosity of the powder bulk of fine particles, it was expected to achieve high relative density and high shape retention of the cubes. All parameters were unchanged from those used for build 4.

Compared to build 4, the negative effects of low energy input could be partially suppressed and the samples no longer contained large inclusions of agglomerates of non-melted particles. Within some specimens, the effects still occurred in correlation to energy input. The changed packing density of the used powder bulk ensured less indication of overheating in comparison to build 4. On the micrographs of the samples that were built up with P_L_ = 120 W and in comparison with low scanning speeds, v_s_ = 50 mm/s, effects were visible that indicated overheating of the melt due to excessive laser power.

**Figure 10:**
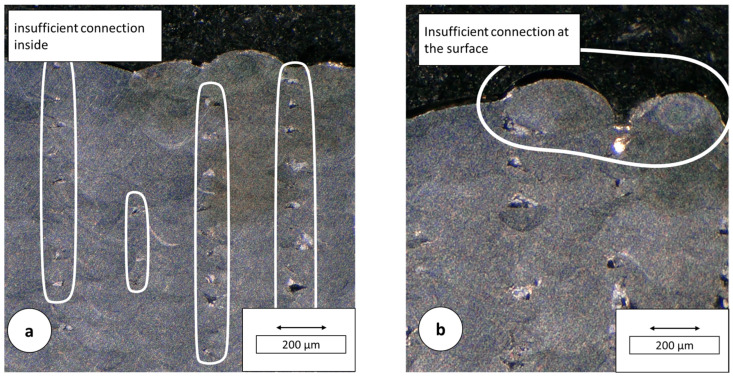
Insufficient connection: specimen from build 5 P_L_ = 120 W, v_s_ = 75 mm/s, Δy_s_ = 300 µm. (**a**) insufficient connection inside; (**b**) Insufficient connection at the surface.

In the etched transverse sections of the specimens from build 5 and 6, complete bonding of the fusion lenses in the vertical direction was observed. In addition, insufficient energy input due to low laser power was observed. The laser powers of 120 W and 150 W, with v_s_ = 75 mm/s, Δy_s_ = 300 µm, used were insufficient to ensure a connection between the individual layers (Figure 10).

In the specimens that were built up with v_s_ = 75 mm/s and v_s_ = 100 mm/s, linear defects were visible again, one above the other. The melt pool spread of these specimens was not wide enough to achieve a sufficient overlap of the tracks, which aligns to the literature [21]. This may indicate an energy input that is too low. The samples, which were built up with a laser power of 150 W, showed a better shape retention and smoother surface than the samples of low laser power, especially for high scanning speeds. In particular, in the range of v_s_ = 50–75 mm/s and y_s_ = 250 μm, the samples showed fine surfaces and few inclusions. As the hatch distance increased, the number of inclusions increased also and the surface pronounce more uneven and wavy. The linear arrangement of the inclusion points to insufficient track overlap as the underlying reason. By increasing the volume energy density, the quantity and size of the inclusions increased. Since the size of the inclusions increased with higher volume energy density despite the expected sufficient coalescence of the particles, we attribute this increase to increased spatter formation as a result of an overheated melt.

The hatch distance can be calculated with the melt pool width (W) and the degree of overlap of two adjacent melt lines/tracks. The melt pool width measured from the etched transverse sections is W = 248.7 µm for build 5 and W = 295.5 µm for build 6. From the data of the optimum degree of overlap of O_r_ = 0.3 (Di et al.) and O_r_ = 0.54 (Dong et al.), the average value can be calculated to be O_r_ = 0.42. This results in an optimum hatch distance for build 5 of Δy_s_ = 144.2 µm [22,23]. For the wider melt pools from build 5, the optimum hatch distance is Δy_s_ = 171.4 µm. 

The guide value according to Meiners [19] leads to a theoretical hatch distance of Δy_s_ = 0.7 × 140 µm = 98 µm for a focal diameter of d_s_ = 140 µm.

**Figure 11:**
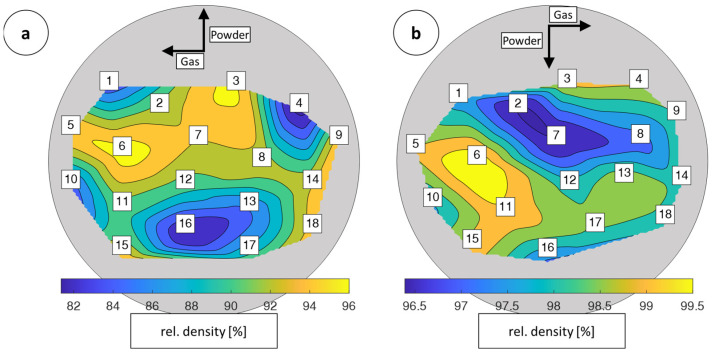
Relative density values of the samples from build 5 MINI (**a**) and build 6 ALPHA (**b**).

The Figure 11a,b shown boxes indicate the arrangement of the individual specimens on the build plates within the MINI and ALPHA. Due to the design of the different machines, the alignment of the coating and the shielding gas flow is rotated by 180° in relation to the positioning of the test specimens.

In Figure 11a, the results from the measurement of the relative density for build 5 on the MINI are shown. The average value was 98.31%. Powder and gas flow are interchanged within the two partial illustrations, because they are arranged differently in terms of the system’s characteristics.

**Figure 12:**
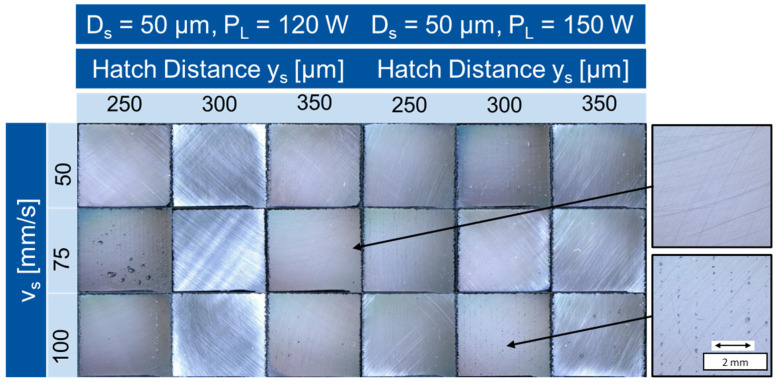
Microscopic images of the samples from build 6 ALPHA.

The first build with the ALPHA, Figure 11b, with a minimum density of 96.48%, specimen number 7, and a maximum density of 99.84%, specimen number 6, indicated a delta of 3.36%. On the microscopic images, which are shown in Figure 12, few pores were observed overall. The pores presented are regularly arranged in the vertical direction. Irregularly arranged pores were not visible.

On the micrographs of the samples that were built up with P_L_ = 120 W and v_s_ = 50 mm/s, effects were clearly visible that indicated overheating of the melt due to excessive laser power. The side surfaces of the samples are very rough and especially the samples built up with a low hatch distance contained many large inclusions. Both are caused by spattering, which occurred more frequently with low viscosity melts and strong evaporation due to overheating of the melt [14].

The wavy shape of the top surface occurred when the degree of overlap of the individual tracks was too high. Therefore, the shape of the top surface became more uniform with increasing hatch distance, relative to the low scan speed samples. However, adjusting the scanning speed was not sufficient. The specimens built with P_L_ = 120 W and v_s_ = 50 mm/s showed how the structure of the surface and the shape retention became finer with increasing hatch distance. However, the samples with a higher hatch distance contained more enclosed un-melted particles [19].

The highest relative density in build 6 was measured at P_L_ = 150 W, v_s_ = 100 mm/s and y_s_ = 250 μm with 96.58%. This specimen also had a uniform surface area and did not collapse. The highest density measured in the entire series of experiments for the cubes made of powder with fine grain content was lower than the density of the cubes without fine grain content. For both laser powers tested, the density of the samples built up with low scanning speed and therefore high-volume energy density were the lowest. The measured values correlate with the microscope images described above.

The lowest densities were measured for the samples built with a high laser power and low scanning speed. This indicated, similar to the observations from the previous paragraphs, that the applied energy was too high for these parameter combinations and consequently defects were more likely to occur.

The influence of the laser beam source and the motion system on the manufacturing result in the PBF-LB/M was investigated. The following observations were made on the low-cost machine compared to a conventional machine when using process-foreign sinter powder:

- On average, the produced samples had higher relative density and a lower standard deviation of the density values.
- The pores present were arranged linearly in the vertical direction.
- The delta between the minimum and maximum relative density of the builds was lower.
- A trend with respect to higher density at higher scanning speed, which was shown on the conventional machine, is not present on the low-cost machine.
- Despite the relatively low scanning speed, samples with a relative density of up to 99.84% could be produced.

## 4. Discussion

### 4.1. Investigation of the Melting Mechanism

For an unambiguous determination of the melt lens geometry, a single exposure must be carried out under otherwise identical conditions to a full build. Thus, the melting mechanism cannot be concluded from the aspect ratio of the melting lenses in the final part, according to Elmer et al. [24]. For this reason, analytical methods from the literature are used.

Due to the high particle diameter and the high internal friction of water atomized particles, the porosity of the powder spill is high. When the laser beam hits the powder, the air-filled spaces are filled by the melt such that the height of the solidified layer decreases significantly compared to the height of the original powder fill.

The model of the irradiated intensity from the field of laser beam welding only requires knowledge of the parameters of the laser beam and is thus suitable for an initial overview. In the build series, laser powers of 120 W and 150 W were used, and the focus diameter was kept constant at 140 µm. This results in volume energy densities of 7.8 × 10^5^ W and 9.744 × 10^5^ W, respectively. These values are within the transition range indicated by Tenbrock et al., where a change from heat conduction to deep welding occurs [18].

For a more detailed investigation of the melting mechanism, the normalized enthalpy model is applied. In Table 4 the process parameters and the resulting normalized enthalpy are shown.

The calculated normalized enthalpy is below the limit of six to eight given in the literature for the occurrence of deep welding. From the model, it can be concluded that heat conduction welding is the main melting mechanism in the tests performed. Larger pores are thus not due to the presence of keyholes. Instead, lack of fusion (LOF) can be determined as the main cause for the pores in the cubes [25].

### 4.2. Influences of the Parameters on the Process

The process parameters determine the morphology and stability of the melt pool and thus fundamentally influence the density of the manufactured components. Therefore, the process parameters must also be considered when discussing the results. In addition, the parameters, especially the maximum laser power and scanning speed, are limited by the laser and the motion system. By studying the melting mechanism, LOF is identified as the main cause of pore formation. In this section, the influences of the process parameters leading to LOF and how they need to be varied to reduce voiding and thus increase the density of the components are discussed.

The hatch distances calculated with both methods are significantly below the set distances of 250–350 µm. From the calculations, a hatch distance between 100 µm and 170 µm can be assumed to be suitable for the process parameters used. The pores in the manufactured samples due to LOF can thus be justified by the hatch distance being set too high.

With increasing laser power, the relative density dropped in some cases, which can be caused by overheating of the molten bath. As the temperature of the melt increases, its viscosity decreases. This leads to the increased evaporation of the melt and splashes, which are induced by the rising gases. The splashes fall back into the powder bed as solidified particles, cannot be completely melted due to their size and remain as inclusions in the material, which aligns to the literature [14]. In particular, build 5 showed that a further increase in the used laser power would introduce more energy into the powder layer, allowing the powder to melt more completely. A further reduction in the scanning speed is not sensible since the build-up of the specimens or parts would take more time and the defect formation would increase. With higher hatch distance, as explained above, no great improvement in the fill level of the samples can be expected either.

### 4.3. Influences of the Laser Beam Source on the Process

The investigations of Grünewald et al. and Wischeropp et al. determined that more stable processes can be achieved with laser beam intensity profiles that deviate from the classical Gaussian profile of single-mode fiber lasers. This is manifested, among other influences, by a lower sensitivity of the relative density to changing process parameters. Multimode fiber lasers or diode lasers thus offer an interesting alternative in the context of low- and best-cost PBF-LB/M concepts [26,27].

The ALPHA samples from build 6 show a significantly higher relative density compared to the MINI samples from build 5. In addition, there are no large or irregularly arranged defect sites in the samples from build 6 and the relative density values within a sample are less scattered. While the relative density of the samples from experiment 1 varies by 15 percentage points, specimens with a relative density between 96.5% and 99.8% can be produced with the same parameter field in build 6. The process on the ALPHA is less sensitive to changing parameters. For this reason, the trend of higher density with increasing scan speed from build 6 is much less pronounced in build 5. These findings supported by the investigations from the literature suggest a higher process stability on the LMI ALPHA 140 compared to the Aconity 3D MINI for the experiments carried out [27].

Another cause of the lower porosity in build 5 is the wider melt pools. For the same hatch distance, wider melt pools result in a higher degree of overlap, so that the occurrence of LOF is minimized. Since the process parameters are not changed between the builds, the different widths of the melt pools can also be justified by the different lasers. This finding is consistent with the studies of Wischeropp et al. who observed wider melt pools with a donut-shaped intensity profile than with a Gaussian profile under the same conditions [27].

Overall, the following advantages of a laser beam source with a multimodal intensity profile can be summarized:

1. More stable process control due to more homogeneous temperature distribution and thus more uniform melt pools with no or only little vaporization of powder.
2. Wider melt pools, allowing larger track gaps.
3. Lower sensitivity regarding the change of process parameters and thus a larger window of stable parameters.

### 4.4. Influences of the Movement System on the Process

**Figure 13:**
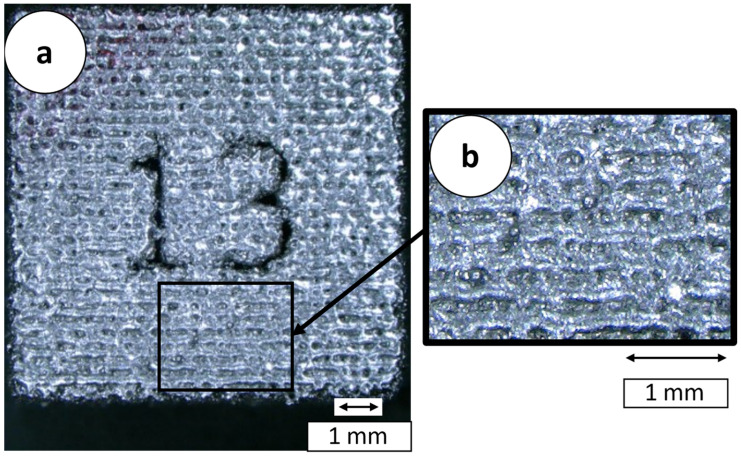
Top view of a specimen from build 3, specimen number 13, P_L_ = 120 W, v_s_ = 50 mm/s, Δy_s_ = 350 µm. (**a**) overview; (**b**) close up.

The too large hatch distance, caused by the positioning accuracy of the gantry, was determined to be the cause of the LOF and thus of the defects within the specimens. In addition to the hatch distance, the motion system affects the maximum scan speed that can be set. The effect of balling due to excessively low scan speeds, which was investigated by Gu and Shen, cannot, or only in isolated cases, be detected in the manufactured samples. The top view of a cuboid, specimen with the number 13, from build 1 is shown in Figure 13. It shows predominantly connected weld lines. The missing connection across the exposure direction is a result of the too large hatch distance [22].

### 4.5. Performance in the Process and Quality of the Components

In the course of this work, it was possible to build up samples from the powder in a stable running process. Changing the parameters as well as adjusting the process cycle resulted in an increase in density and better shape retention of the specimens. The density values measured are above the values previously achieved in other studies on comparable powders in the PBF-LB/M process. The highest density achieved is 99.84%, which is 0.16% below the density achieved for PBF-LB/M components made of 1.4404 [28]. The highest relative density of the builds performed is achieved with the parameters P_L_ = 120 W, v_s_ = 100 mm/s, y_s_ = 250 µm, d_s_ = 140 µm and D_s_ = 50 µm. The parameter range in which components of high density can be built up was narrowed down in terms of the scanning speed and hatch distance.

Despite shorter build times for builds with high scan speeds and hatch distances, high density values are also achieved for both laser powers in the builds. Depending on the application and post-processing possibilities, these parameter combinations are of interest. A similar trend is also visible in the builds with powder without fine grain content, but the density values achieved were lower and the specimens contain larger, partially sharp-edged inclusions, which can negatively influence the mechanical stress ability.

## 5. Conclusions

The work sets an incentive for further research in the area of best cost concepts in PBF-LB/M, as well as in the processing of coarse-grained sinter powders in the PBF-LB/M process.

From the observations, the following conclusions are drawn regarding the use of a multimode rather than a single-mode fiber laser, each supported by findings from the literature:

- Eliminating irregularly distributed pores indicates a process with more stable weld pools. The number of pores between each weld path is smaller due to wider melt pools.
- The process is less sensitive to changing process parameters, resulting in a wider window of stable parameters.

A gantry system has proven suitable for use in PBF-LB/M, despite the lower scanning speed. Beam guidance in the XY-plane introduces new possibilities for machine and process design that are not possible with conventional scanner systems.

A fiber laser was used in this series of experiments. Other lasers have a more homogeneous intensity distribution, which can affect melt pool behavior and melt pool expansion. It is expected that other laser media and further adjustment of the laser focus can improve the quality of the components.

Experimental as well as numerical models comparing different coater designs and their effect on the porosity of a powder bed have shown that a powder roller can generally achieve a lower porosity in the powder bed than a slider and would thus be a cost-effective way to increase the relative density of the components and may even eliminate the double coating and exposure procedure [9].

### Future Outlook

The main challenge is the complete melting of the powder particles. In terms of materials costs and the particular application, it should be examined how great the influence of inclusions of non-melted powder particles is on the mechanical properties of the components built up from them. Tensile tests and hardness measurements can provide information on whether the mechanical properties meet the requirements of the application.

In order to compare the melt pool morphology more accurately, individual exposures must be performed on both machines. To shorten the build time, the behavior at higher scan speeds and higher laser power must be investigated. In order to exclude further influences of the machine, a machine is needed that allows an exchange of the process parameters on the individual specimens under investigation under otherwise identical conditions.

Some innovations related to beam steering in the XY-plane have been presented, and more are expected in the future. The applicability of these ideas needs to be explored.

## Figures and Tables

**Table 1:** Chemical composition Distaloy HP1, manufacturer’s data [11].

Powder	Mo [%]	Ni [%]	O [%]	C [%]	Cu [%]	Cr [%]	Fe [%]
Distaloy HP1	1.41	4.00	0.008	<0.01	2.00	0	Res.

**Table 2:** Predefined process parameters based on low-cost machine equipment.

Parameter	Minimum	Maximum
Laser power [W]	-	150
Spot diameter [µm]	100	200
Wavelength [nm]	976 ± 7 nm (diode)–1030–1080 nm (fiber)	
Scanning speed [mm/s]	50	500
Acceleration [m/s^2^]	10	10
Repeatability [mm]	0.3	0.3

**Table 3:** Variations within the individual builds.

Build [#]	PSD	P_l_ [W]	D_s_ [µm]	v_s_ [mm/s]	d_s_ [µm]	y_s_ [µm]	Ex&Co	Machine
1	45–90	120	100	100–300	76	50–150	1×	MINI
2	45–90	120	50	100–300	140	50–150	1×	MINI
3	45–90	120/150	50	250–350	140	50–150	1×	MINI
4	45–90	120/150	50	250–350	140	50–150	2×	MINI
5	0–90	120/150	50	250–350	140	50–100	1×	MINI
6	0–90	120/150	50	250–350	140	50–100	1×	ALPHA

**Table 4:** Process parameters and the resulting normalized enthalpy.

Laser Power PL [W]	Scanning Speed v_s_ [mm/s]	Focus-Ø ds [µm]	Normalized Enthalpy ∆H [kg m^2^/s^2^]
120	50	140	3.676
	75		3.001
	100		2.599
150	50		4.595
	75		3.752
	100		3.249
